# Supplementary material for: Splice-Junction-Based Mapping of Alternative Isoforms in the Human Proteome
Source: Cell Rep. Author manuscript; Available in PMC 2020 Jan 15. (PMC6961840; doi:10.1016/j.celrep.2019.11.026)
Supplement: 2 [file NIHMS1546469-supplement-2.zip › DF1/PXD006675/RightVentricle/RightVentricle_38_YWHAZ_TQGDEAEAGEGGERFGVSYPGIVDQSQQAYQEAFEISK.pdf]

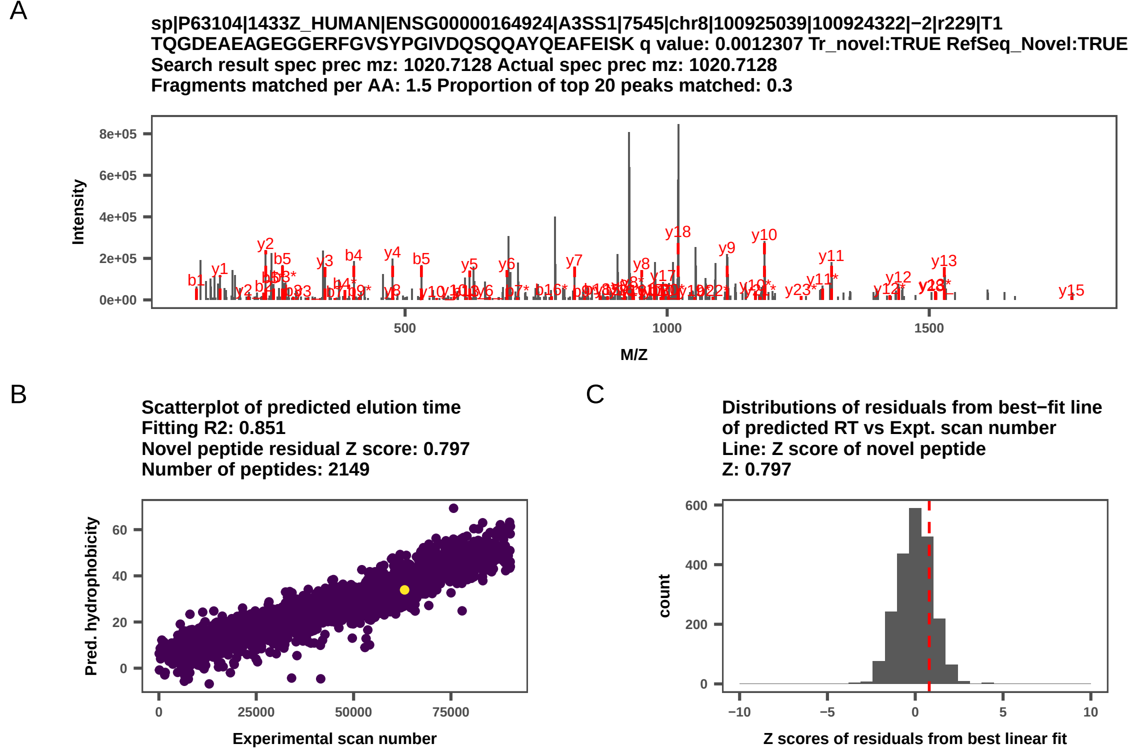

A
sp|P63104|1433Z_HUMAN|ENSG00000164924|A3SS1|7545|chr8|100925039|100924322|−2|r229|T1
TQGDEAEAGEGGERFGVSYPGIVDQSQQAYQEAFEISK q value: 0.0012307 Tr_novel:TRUE RefSeq_Novel:TRUE
Search result spec prec mz: 1020.7128 Actual spec prec mz: 1020.7128
Fragments matched per AA: 1.5 Proportion of top 20 peaks matched: 0.3
Intensity
8e+05
6e+05
4e+05
2e+05
0e+00
y18
y10
y9
y11
y13
y2
b5
y3
b4
y4
b5
y5
y6
y7
y8
y17
y12
y1
b1
y15
M/Z
500
1000
1500
B
Scatterplot of predicted elution time
Fitting R2: 0.851
Novel peptide residual Z score: 0.797
Number of peptides: 2149
Pred. hydrophobicity
60
40
20
0
0
25000
50000
75000
Experimental scan number
C
Distributions of residuals from best−fit line
of predicted RT vs Expt. scan number
Line: Z score of novel peptide
Z: 0.797
count
600
400
200
0
−10
−5
0
5
10
Z scores of residuals from best linear fit
